# Nutritional Status Is Associated with Health-Related Quality of Life, Physical Activity, and Sleep Quality: A Cross-Sectional Study in an Elderly Greek Population

**DOI:** 10.3390/nu15020443

**Published:** 2023-01-14

**Authors:** Sousana K. Papadopoulou, Maria Mantzorou, Gavriela Voulgaridou, Eleni Pavlidou, Konstantinos Vadikolias, Georgios Antasouras, Theofanis Vorvolakos, Evmorfia Psara, Georgios K. Vasios, Aspasia Serdari, Efthymios Poulios, Constantinos Giaginis

**Affiliations:** 1 Department of Nutritional Sciences and Dietetics, School of Health Sciences, International Hellenic University, 57400 Thessaloniki, Greece; 2 Department of Food Science and Nutrition, School of Environment, University of Aegean, 81400 Myrina, Lemnos, Greece; 3 Department of Neurology, School of Medicine, Democritus University of Thrace, 68100 Alexadroupoli, Thrace, Greece; 4 Department of Geriatric Psychiatry, School of Medicine, Democritus University of Thrace, 68100 Alexadroupoli, Thrace, Greece; 5 Department of Psychiatry and Child Psychiatry, School of Medicine, Democritus University of Thrace, 68100 Alexadroupoli, Thrace, Greece

**Keywords:** nutritional status, health-related quality of life, physical activity, sleep quality, public health, Body Mass Index

## Abstract

This study aims to explore the associations between nutritional status and health-related quality of life, physical activity, and sleep quality in older exclusively Caucasian adults from Greec who were free of any severe disease. This is a cross-sectional study. Mini Nutritional Assessment was used to assess nutritional status, health-related quality of life was assessed using the Short Form Healthy Survey questionnaire, sleep quality was assessed using the Pittsburgh Sleep Quality Index, and physical activity levels were assessed via the International Physical Activity Questionnaire. 3405 community-dwelling men and women, over 65 years old from14 different Greek regions were enrolled. Ten-point four percent (10.4%) of the participants were classified as malnourished, while 35.6% were “at risk of malnutrition”. A better nutritional status was significantly and independently associated with higher physical activity levels (*p* = 0.0011) and better quality of life (*p* = 0.0135), as well as better sleep quality (*p* = 0.0202). In conclusion, our study highlights the interrelationships between a good nutritional status, a high-quality sleep, active lifestyle, and good quality of life. Further interventional studies are needed to clarify the associations, and test the feasibility of improving the nutritional status, physical activity levels and sleep quality of the elderly, and the impact of these changes on quality of life, and healthy ageing in races beyond Caucasian populations. Public health strategies and policies should be recommended to inform older adults for the necessity to improve their nutritional status and lifestyle habits to improve their health status and to obtain better life expectancy.

## 1. Introduction

Population ageing has put healthy ageing in a position of global priority (World Health Organization, [1]. As the population is getting older, the need for better disease prevention and management is more prevalent. According to the Pan American Health Organization (2021) “Healthy aging is a continuous process of optimizing opportunities to maintain and improve physical and mental health, independence, and quality of life throughout the life course” hence we need to focus on a more inclusive perspective regarding ageing, that takes into account both the physical and mental aspects of being, and associated factors [2].

There is substantial evidence that the elderly constitutes a population group with a high prevalence of non-communicable chronic diseases and high risk of malnutrition in Europe [3]. According to the recoded score of the ‘Determine your nutritional health’ (NSI checklist), 53% of the elderly were at nutritional risk in Europe [3]. The exiting evidence has emphasized unfavourable consumptions of total and saturated fat, sugar, salt and dietary fibre together with low intakes and suboptimal status of key micronutrients such as vitamins D, B2, B12, folate and calcium in European older adults’ populations [4].

Living with a good quality of life is a crucial factor for the wellbeing of the elderly [1,2] and it is not just living with the absence of disease, but having a meaningful life and maintaining relationships, feeling well even when living with a disease [5] and being physically healthy and independent [6]. With the population ageing and the prevalence of chronic diseases [7], the assessment and improvement of the life quality of the elderly is key area of public health interest.

Nutritional status also plays a key role on healthy ageing [8,9]. Diet quality and quantity are declining in this age group, making the elderly a group at high risk of malnutrition [8,10,11] and mortality [11], endangering health status [9,12,13,14] and quality of life [13]. In fact, malnutrition in this age group can have both physical health consequences, such as increased risk of falls, and frailty [15,16,17] and mental health consequences, in regard to depression and cognitive decline [9,18]

Being physically active is key for healthy ageing, with studies and meta-analyses con-firming the integral role of physical activity [19,20]. In fact, one sixth of deaths in the UK can be attributed to physical inactivity [21], while the World Health Organization guidelines suggest that the elderly should engage in at least “150–300 min of moderate-intensity aerobic physical activity” and “as part of their weekly physical activity, older adults should do varied multicomponent physical activity that emphasizes functional balance and strength training at moderate or great-er intensity, on 3 or more days a week, to enhance functional capacity and to prevent falls”. Physical activity has been associated with better physical and mental health status, and better quality of life in the elderly [22,23], as well as with longevity [24]. Being physically active is safe for the elderly, even those with frailty. Regular physical activity is recommended due to its health benefits regarding cardiometabolic health, cognitive status, and musculoskeletal health [25].

However, the currently available data suggest that the elderly do not engage in physical activity [25] and this lifestyle choice can lead to health implications [26] and higher mortality rates [27,28]. In fact, physical activity can improve depressive symptoms and quality of life in institutionalized elderly persons [29], and it can be used to prevent falls in this age group [30].

Sleep is another important physiological function of the body, which has not been ad-equitably explored, while the past years, studies have demonstrated that sleep quality is an important factor for physical and mental health [31]. Regarding the elderly, they are an age group with high risk of sleep disorders [32]. Long and short sleep has been associated with chronic disease, with data indicating that short sleep duration is associated with type II diabetes and glucose metabolism dysregulation [33], as well as cardiovascular disease [34], while sleep longer than 9 h has been associated with higher risk of cardiovascular disease, type 2 diabetes, depression, obesity, and chronic kidney disease [35].

In view of the above considerations, the present study aims to explore the associations between nutritional status, health related quality of life, physical activity levels and sleep quality in Greek Caucasian older adults from 14 different Greek regions who did not suffer from any severe disease.

## 2. Materials and Methods

### 2.1. Subjects

Initially 6745 community-dwelling Greek Caucasian older adults were randomly enrolled from 14 different Greek regions, both urban, rural and islands, namely Athens, Thessaloniki, Alexandroupoli, Kavala, Ioannina, Larissa, Lamia, Korinthos, Patra, Tripoli, Kalamata, Crete, North and South Aegean. Recruitment to the study was between April 2014 and December 2019 in community-dwelling older adults founding mainly during their visit in health care units, as well as in public centers related with entertainment activities for older persons.

During their thorough recruitment, older adults that had no severe, untreated, chronic disease symptoms such as any cardiovascular disease, any cancer or premalignant disease, metabolic disorders, autoimmune diseases, or neurodegenerative diseases were not included in the study. Finally, 3405 older adults were included in the study with a final response rate equal to 50.4%. The study was conducted according to the guidelines of the Declaration of Helsinki and in accordance with the World Health Organization (52nd WMA General Assembly, Edinburgh, Scotland, 2000). All procedures involving research study participants were approved by the Ethics Committee of the University of Aegean.

### 2.2. Study Design

Mini Nutritional Assessment (MNA), a validated questionnaire to use in elderly per-sons geriatric assessment [36,37], was used to assess the nutritional status of the participants. Trained physicians (e.g., medical and nursing personnel and nutritionists and dietitians) explained in detail to the community-dwelling older adults the questions of the questionnaires to facilitate accurate answers. We performed a face-to-face interview where each interviewer directly communicates with the respondent in accordance with the prepared questionnaires. Trained nutritionists, dietitians and physicians measured anthropometric indices, Body Mass Index (BMI), mid arm circumference and calf circumference as per protocol [9,38]. Older adults’ weight was measured using a Seca scale [Seca, Hanover, MD], without shoes, to the nearest 100 g while height was measured using a portable stadiometer (GIMA Stadiometer 27335) with no shoes on, to the nearest 0.1cm. Two international datasets were used to define overweight and obesity in pre-school children and their mothers: the WHO and the International Obesity Task Force (IOTF) data references [39,40].

We used three validated questionnaires to evaluate health-related quality of life, physical activity levels and sleep quality of the study population, respectively. Specifically, we assessed Health-Related Quality of Life (HRQOL) utilizing the Short Form Healthy Survey (SF-36) questionnaire [41]. The SF-36 questionnaire consists of 36 items. All but one item are assigned to one of the eight health domains covering various aspects of physical and mental health: physical functioning (PF, 10 items), physical role functioning (RP, 4 items), bodily pain (BP, 2 items), general health perceptions (GH, 5 items), vitality (VT, 4 items), social role func-tioning (SF, 2 items), emotional role functioning (RE, 3 items), and mental health (MH, 5 items) [41]. For these subscales, a score ranging from 0 (worst) to 100 (best) was calculated.

Physical activity levels were assessed using the International Physical Activity Questionnaire (IPAQ) in which subjects mention how much exercise they did in a typical week. This self-administered questionnaire, used worldwide, assesses the overall physical activity over the last seven days, to categorize it as low, moderate, or high. IPAQ instruments have been tested in both developed and developing countries and demonstrated good reliability and acceptable validity properties, at least as good as other self-answered PAQs [42]. Briefly, the purpose of IPAQ-Gr is to sum up vigorous, moderate, and walking PAs over the previous seven-day period and generate a total physical activity score (PAscore), expressed in MET-minutes per week (MET.min.wk^−1^). Based on the IPAQ scoring procedure, PA status is classified into three categories (PAclasses): (1) low PAclass, insufficiently active subjects (total PAscore < 600 MET.min.wk^−1^); (2) moderate PAclass; and (3) high PAclass, HEPA active subjects, (HEPA: health-enhancing physical activity, i.e., total PAscore ≥ 3000 MET.min.wk^−1^ or vigorous PAscore ≥ 1500 MET.min.wk^−1^ [42]. The questionnaires were completed by trained physicians (e.g., medical and nursing personnel) and nutritionists and dietitians during one-to-one interviews with community-dwelling older adults.

Sleep quality was assessed using the Pittsburgh Sleep Quality Index (PSQI) [43,44] which consists of 19 items which are rated on a four-point scale (0–3) and grouped into seven components (sleep quality, sleep latency, sleep duration, habitual sleep efficiency, sleep disturbance, use of sleeping medications, and daytime dysfunction). The item scores in each component were summed and converted to component scores ranging from 0 (better) to 3 (worse) based on guidelines. Total PSQI score were calculated as the summation of seven component scores ranging from zero to 21, where higher score indicates worse condition. A total global PSQI score of <5 is indicative of adequate sleep quality [43,44].

Clarifying instructions were given to the participants about the completion of questionnaires, and a detailed presentation of the questions to facilitate accurate answers was per-formed. Anthropometric indices were measured by trained nutritionists and physicians as per protocol [39]. Weight was measured using the same electronic scale, and height was measured using a portable stadiometer. A non-elastic measuring tape was used to measure the mid-arm and calf circumference.

Sample size calculation was based on the use of PS: Power and Sample Size calculator programme, while the randomization was carried with the use of a sequence of random binary numbers (e.g., 001,110,110 in which 0 represented enrolment and 1 not enrollment to the study). The questionnaires were completed by trained nutritionists and physicians during one-to-one interviews with the participants.

Economic level, living status and smoking habits were retrieved by the questionnaires. Financial status was classified according to the annual income as: 0 ≤ 5000€, 1 ≤ 10,000€, 2 ≤ 15,000€, 3 ≤ 20,000€, 4 ≤ 25,000€ and 5 > 25,000€. Financial status was further classified as low for annual income ≤ 10,000€, medium for annual income > 10,000€ and ≤20,000€, and high for annual income > 20,000€. Education level was assessed, as years of education. Living status was classified as living completely alone or living with others, i.e., husband or wife or their children or other relatives. Elderly participants were further classified as never smokers and smokers.

### 2.3. Statistical Analysis

Statistical analysis was performed by Student’s t-test and one-way ANOVA for continuous variables found to follow the normal distribution by the use of Kolmogorov-Smirnov test. All continuous variable followed norm distribution after Kolmogorov-Smirnov testing. Chi-square test was used for categorical variables. The normally distributed quantitative variables are presented as mean value ± Standard Deviation (SD), and the qualitative variables as absolute or relative frequencies. Multiple logistic regression analysis was performed to assess the influence of nutritional status in health-related quality of life, physical activity levels and sleep quality after adjustment for possible confounders. Differences were considered significant at *p* < 0.05 and 95% Confidence Interval. The statistical analysis of the survey data was performed by Statistica 10.0 software, Europe (In-former Technologies, Inc., Hamburg, Germany).

## 3. Results

### 3.1. Sociodemographic and Anthropometric Characteristics and Nutritional Status Evaluation

Descriptive statistics of the study population are presented in Table 1. The mean age of study population was 74.6 ± 8.3 years old. Concerning participants’ gender, 48.6% were female and 51.4% were men. The mean BMI was 27.3 ± 4.1 kg/m^2^. According to BMI classification, 46.8% of the participants had normal weight, 39.2% were overweight and 14.0% were obese. 74.6% of elderly had mid arm circumference ≥22 cm and 25.4% of participants had mid arm circumference <22 cm. 65.3% of elderly had calf circumference ≥31 cm and 34.7% of participants had calf circumference <31 cm.

The mean value of the educational years was 7.3 ± 2.8 years. Regarding the economic status, 42.9% of the elderly reported low annual income, 38.3% medium and 18.8% high annual income. Concerning the living status, 34.9% of the elderly were living alone and 65.1% were living with others. With regard to the smoking habits, 24.2% were smokers and 75.8% were never smokers.

Nutritional status was assessed by MNA. MNA Score variable was normally distributed-ed according to Kolmogorov-Smirnov test. The mean value for MNA score was 23.5 ± 4.2 points (minimum = 6.5-maximum = 30). Participants with score ˂ 17 (10.4%) were classified to present malnutrition, while those with scores at 17–23.5 points (35.6%) were at risk of malnutrition. Participants with scores ≥ 24 (54.0%) were pointed to be well nourished.

### 3.2. Health-Related Quality of Life, Physical Activity, and Sleep Quality Evaluation

Health-related quality of life (HRQOL), physical activity (IPAG), and sleep quality evaluation (PSQI) variables was non-normally distributed according to Kolmogorov-Smirnov test. As far as HRQOL questionnaire, the median score was 53/100 (IQR: 38–73). More to the point, 50,1% of elderly had low health-related quality of life (HRQOL, score ˂ 53/100), while 49.9% had high health-related quality of life (HRQOL, score ≥ 53/100) after dichotomization according to the median value.

Considering the physical activity levels (IPAQ) of the study population, 41.0% had low physical activity levels, 34.4% had moderate physical activity levels and 24.6% had high physical activity levels. Regarding Pittsburgh Sleep Quality Index (PSQI) of the study population, 56.9% of elderly had adequate sleep quality and 43.1% had inadequate sleep quality.

### 3.3. Comparisons between Nutritional Status and Sociodemographic and Anthropometric Characteristics

In crosstabulation, nutritional status was significantly better in younger, than older participants (Table 2). According to BMI classification, malnourished participants, as well as those at risk for malnutrition were significantly more frequently overweight or obese than well-nourished participants (Table 2). The incidence of participants with mid arm circumference <22 cm, an indication of low muscle mass, was significantly increased in malnourished participants and those at risk for malnutrition compared to well-nourished participants (Table 2). Accordingly, participants with calf circumference <31 cm, had significantly worse nutritional status than those with calf circumference ≥31 cm (Table 2).

Nutritional status was significantly, positively associated with participants’ educational level and economic status (Table 2). Elderly living with others had significantly better nutritional status than those living alone (Table 2). Participants’ gender and smoking habits were not associated with nutritional status (Table 2).

### 3.4. Comparisons between Nutritional Status and Health-Related Quality of Life, Physical Activity, and Sleep Quality

In crosstabulation, participants with worse nutritional status had significantly more frequently lower HRQOL scores compared to well-nourished ones (Table 2). Well-nourished participants had significantly more frequently moderate or high physical activity levels compared to those at risk of malnutrition and malnourished ones (Table 1). Well-nourished participants also had significantly more frequently adequate PSQI compared to those at risk of malnutrition and malnourished ones (Table 2).

**Figure 1:**
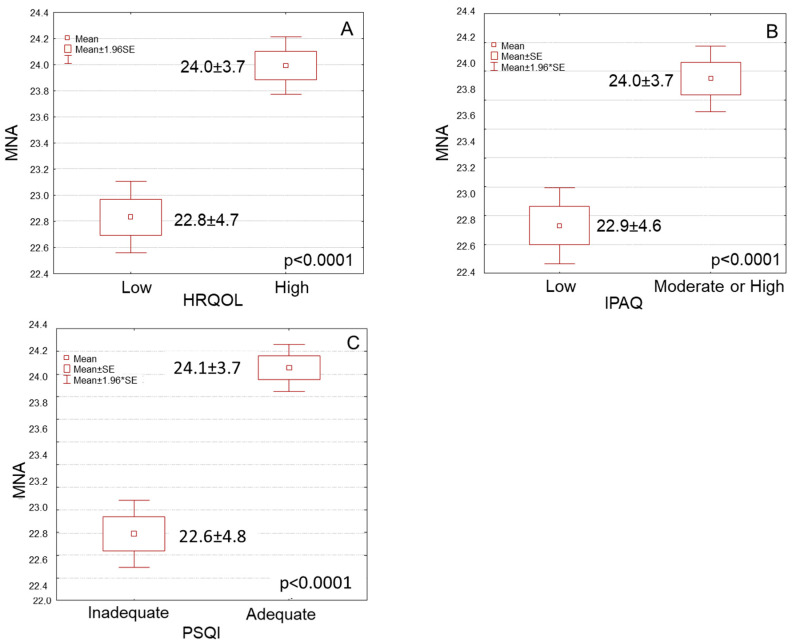
Box and whisker plots for nutritional status according to MNA of elderly participants in association with their (**A**) HRQOL, (**B**) IPAQ and (**C**) PSQI.

Using nutritional status as continuous variable, participants with high HRQOL had significantly higher mean MNA scores compared to those with low HRQOL (Figure 1A, 24.0 ± 3.7 vs. 22.8 ± 4,7, *p* ˂ 0.0001). Participants with moderate or high physical activity levels also had significantly higher mean MNA scores compared to those with low physical activity levels (Figure 1B, 24.0 ± 3.7 vs. 22.9 ± 4.6, *p* ˂ 0.0001). Moreover, participants with adequate sleep quality had significantly higher mean MNA scores compared to those with inadequate sleep quality (Figure 1C, 24.1 ± 3.7 vs. 22.6 ± 4.8, *p* ˂ 0.0001).

### 3.5. Multiple Regression Analysis for Mediterranean Diet Adherence after Adjustment for Potential Confounding Factors

In multiple logistic regression analysis, nutritional status was independently associated with HRQOL, physical activity and sleep quality, after adjustment for potential confounding factors (Table 3, *p* = 0.0011, *p* = 0.0135 and *p*= 0.0202, respectively). Specifically, well-nourished elderly had a 2.1-fold better HRQOL than those at risk or malnutrition or mal-nourished ones (Table 3, *p* = 0.0011). Moreover, well-nourished elderly had an 82% higher probability of having moderate or high physical activity levels than those at risk or malnutrition or malnourished ones (Table 3, *p* = 0.0135). Well-nourished participants had also a two-fold better sleep quality than those at risk or malnutrition or malnourished ones (Table 3, *p* = 0.0202). Participants’ age, BMI, and mid arm and calf circumference were also independently associated with nutritional status (Table 3, *p* ˂ 0.05). In fact, overweight and obese older adults showed an 81% higher probability of worse nutritional status (*p* = 0.0045). Older adults with mid arm circumference <22 cm had a 29% higher likelihood of worse nutritional status (*p* = 0.00007), while participants with calf circumference <31 cm presented an 18% higher risk of worse nutritional status (*p* = 0.0004).

## 4. Discussion

Factors that impact the wellbeing and functionality of the elderly play a significant role on ensuring the successful ageing and life expectancy of older aged population. Hence, we aimed to explore the associations between nutritional status, physical activity, quality of life, and the less explored sleep quality in a sample of 3405 community-dwelling exclusively Caucasian Greek older adults, men and women who were diseases-free concerning untreated sever disease.

In our study, 10.4% of the participants were classified as malnourished, while 35.6% were “at risk of malnutrition”. “Malnourished” and “at risk for malnutrition” participants, when compared to “well-nourished” participants, were older, had lower BMI, and lower mid arm circumference indicating lower muscle mass. Malnutrition in the elderly is quite prevalent, and ranges between 4–30%, dependent on the setting [1,9]. The high prevalence of poor nutritional status in our study participants raises a serious public health concern, as discussed in our previous publication [9]. In line with other studies, older age [45] is a risk factor for malnutrition, while loss of muscle mass is indicative of malnutrition [46,47]. The loss of muscle is in accordance with our study concerning the increased prevalence of low mid arm and circumference of our study population.

As expected, and according to the literature [25] only one in four of the participants had high physical activity levels, while just over half of the participants had an adequate sleep quality (56.9%) [48]. Our analysis showed that a better nutritional status, according to the MNA was significantly and independently associated with higher physical activity levels (IPAQ) and better quality of life (SF-36), as well as better sleep quality (PSQI) in an exclusively Caucasian population from Greece. In elderly peritoneal dialysis patients, malnutrition-inflammation score was a prognostic factor for physical activity levels, while body composition was also associated with physical activity [49]. Moreover, a 12-week intervention with oral nutritional supplements and exercise in frail institutionalized elderly found positive effects on both nutritional status and quality of life [50]. Notable, several studies have indicated that physical activity and sports and exercise therapy programs are not only practicable but also recommendable for oncologic patients during the acute phase and in the aftercare [51].

Verlaan et al. [52] found that in elderly without malnutrition, those with sarcopenia had lower levels of physical activity and lower quality of life. A Spanish study, however, in healthy, physically active and independent elderly did not find a statistically significant association between nutritional status and physical activity but did confirm the association between nutritional status and psychosocial wellbeing [53]. On the other hand, a reactive and pro-active application of muscle-targeted nutritional therapy appears promising and should be proposed [54]. However, future research should be directed toward the management of patient populations characterized by substantial muscle wasting, as these have been frequently excluded from previous trials, perhaps to avoid confounding [54].

Regarding the associations between nutritional status and quality of life, a recent systematic review and meta-analysis of cross-sectional and quasi-experimental studies on the associations between nutritional status and quality of life in institutionalized elderly, found a positive association between those parameters, while noting that nutritional interventions did improve quality of life [55]. Another meta-analysis by Rasheed et al. [56]. that included cohort and interventional studies found that malnutrition is negatively associated with quality of life in the elderly. Further studies also confirm our finding. In Iranian older adults, nutritional status was associated with quality of life, measured by the SF-36 questionnaire [57], while in older adults with rheumatoid arthritis malnutrition was associated with lower quality of life and frailty was significantly independently associated with lower quality of life, as assessed by the WHOQoL-BREF questionnaire [58]. Furthermore, Rasheed and Woods in their study using the EQ-5D and the SF-36 to assess quality of life, they found that poor nutritional status (via MNA-SF) is associated with lower quality of life in hospitalized elderly [59], while in very old Chinese elderly, better health related quality of life, according to the EQ-5D, was independently as-sociated with higher BMI and better sleep quality [60].

Few other studies explore the seemingly bi-directional [61] association between sleep quality and nutritional status in this age group. Findings from the WCHAT study in 6792 middle aged and elderly adults from China, showed that poor sleep quality (assessed by the PSQI) was associated with higher risk of malnutrition (assessed by MNA-SF), while both long and short sleep duration were associated with higher odds of malnutrition [62]. Findings from The Helsinki Businessmen Study [63] also revealed that nutritional status (MNA-SF) was associated with better self-reported sleep quality [63]. Moreover, a study with atrial fibrillation patients, with mean age 68 years, found that as sleep quality worsened (according to PSQI), malnutrition risk (according to MNA) increased [64]. Moreover, nutritional interventions may improve sleep quality in people with intellectual disabilities [65]. Dietary patterns seem to be an important factor to improving the quality and quantity of sleep. However, the current literature regarding the benefit of improved nutrition on sleep in people with an intellectual disability needs to be interpreted with caution [65].

Our study is a cross-sectional study, and to our knowledge, the first study to explore the relationship between nutritional status, health-related quality of life, sleep quality and physical activity in a Greek elderly population. Our study strength is the relatively large and representative study population since it included mothers and their matched children from 14 geographically diverse areas of Greece, urban, rural and island regions. However, the study sample was enough large and includes Caucasian population who live in 14 geographically diverse regions, urban, rural and islands, and therefore its representativeness could be considered as enough high. Thus, our results could be generalizable beyond the Greek population in other Caucasian populations of other ethnicities. However, future studies should be performed on other races which may have several differences concerning genetic background, lifestyle factors, sociodemographic factors. Additionally, we used the validated PSQI questionnaire in order to assess sleep quality. However, due to the fact that it is an observational study, we can only extract associations, and not causation, in our findings. Additionally, despite a thorough approach to confounding adjustment, we acknowledge the possibility for unmeasured confounding. Even though we have adjusted for age, gender, education, economic status, smoking habits, and living status, it is still possible that residual confounding may have affected our results.

## 5. Conclusions

Healthy ageing is an integral part of public health priorities and comprises both physical and mental health aspects. Our study highlights the interrelationships between a good nutritional status, a high-quality sleep, active lifestyle, and good quality of life in Caucasian older adults from Greece which were diseases-free. It is important to focus on these parameters and integrate them into preventative actions against chronic disease, as well as into interventions aiming to improve the health and wellbeing of the elderly. Interventional studies are strongly recommended in order to test the feasibility of improving the nutritional status, physical activity levels and sleep quality of the elderly, and the impact of these changes on quality of life, and healthy ageing. Public health strategies and policies are strongly required to advise older adults for the necessity to improve their nutritional status and lifestyle habits in order to improve their health status and to obtain better life expectancy.

## Figures and Tables

**Table 1:** Descriptive statistics of the study population.

Characteristics, *n* = 3405	Descriptive Statistics
Mean age (years ± SD)	74.6 ± 8.3
Gender (*n*, %)	
Female	48.6%
Male	51.4%
Mean BMI (kg/m^2^)	27.3 ± 4.1
BMI status	
Normal weight	46.8%
Overweight	39.2%
Obese	14.0%
Mid arm circumference (*n*, %)	
<22 cm	25.4%
≥22 cm	74.6%
Calf circumference (*n*, %)	
<31 cm	34.7%
≥31 cm	65.3%
Educational level (years ± SD)	7.3 ± 2.8
Financial status (*n*, %)	
Low	42.9%
Medium	38.3%
High	18.8%
Living status (*n*, %)	
Alone	34.9%
With others	65.1%
Smoking habits (*n*, %)	
Never smokers	75.8%
Smokers	24.2%
HRQOL (*n*, %)	
Low	50.1%
High	49.9%
IPAQ (*n*, %)	
Low	41.0%
Moderate	34.4%
High	24.6%
PSQI (*n*, %)	
Inadequate	43.1%
Adequate	56.9%

**Table 2:** Comparisons between nutritional status and participants’ sociodemographic characteristics, anthropometric measures, HRQOL, IPAQ and PSQI categories.

Characteristics, *n* = 3405	Nutritional Status			
	Malnourished (10.4%)	At Risk of Malnutrition (35.6%)	Well Nourished (54.0%)	*p*-Value
Age (years ± SD)	80.9 ± 7.7	74.9 ± 8.5	73.2 ± 7.8	*p* < 0.0001
Gender (*n*, %)				*p* = 0.0722
Female	158 (44.8)	705 (58.1)	791 (43.0)	
Male	195 (55.2)	509 (41.9)	1047 (57.0)	
BMI status				*p* = 0.0001
Normal weight	119 (33.6)	551 (45.5)	923 (50.2)	
Overweight	148 (41.8)	421 (34.7)	765 (41.6)	
Obese	87 (24.6)	240 (19.8)	151 (8.2)	
Mid arm circumference (*n*, %)				*p* ˂ 0.0001
<22 cm	272 (77.0)	483 (39.8)	109 (5.9)	
≥22 cm	81 (23.0)	731 (60.2)	1729 (94.1)	
Calf circumference (*n*, %)				*p* ˂ 0.0001
<31 cm	291 (82.4)	596 (49.1)	293 (15.9)	
≥31 cm	62 (17.6)	618 (50.9)	1545 (84.1)	
Educational level (years ± SD)	6.6 ± 2.0	7.2 ± 2.7	7.6 ± 3.2	*p* = 0.0002
Financial status (*n*, %)				*p* = 0.0001
Low	195 (55.2)	589 (48.5)	676 (36.8)	
Medium	116 (32.9)	449 (37.0)	738 (40.1)	
High	42 (11.9)	176 (14.5)	424 (23.1)	
Living status (*n*, %)				*p* = 0.0003
Alone	158 (44.8)	423 (34.8)	606 (33.0)	
With others	195 (55.2)	791 (65.2)	1232 (67.0)	
Smoking habits (*n*, %)				*p* = 0.1237
Never smokers	286 (81.0)	927 (76.4)	1367 (74.4)	
Smokers	67 (19.0)	287 (23.6)	471 (25.6)	
HRQOL (*n*, %)				*p* = 0.0002
Low	205 (58.1)	638 (52.5)	859 (46.7)	
High	148 (41.9)	576 (47.5)	979 (53.3)	
IPAQ (*n*, %)				*p* < 0.0001
Low	193 (54.7)	568 (46.8)	636 (34.6)	
Moderate	113 (32.0)	407 (33.5)	649 (35.3)	
High	47 (13.3)	239 (19.7)	553 (30.1)	
PSQI (*n*, %)				*p* = 0.0069
Inadequate	157 (44.5)	569 (46.9)	740 (40.3)	
Adequate	196 (55.5)	645 (53.1)	1098 (59.7)	

Chi-square test for categorical variables and ANOVA test for normally-distributed variables were used.

**Table 3:** Multiple logistic regression analysis for nutritional status after adjustment for potential confounding factors.

Parameters	HR (95% CI)	*p*-Value
Age (Below/Over mean value)	0.76 (0.45–1.12)	0.0012
Gender (Male/Female)	1.33 (0.87–1.98)	0.0839
BMI (Normal/Overweight or Obese)	1.81 (1.53–2.18)	0.0045
Mid arm circumference (<22 cm/≥22 cm)	0.71 (0.45–1.12)	0.0007
Calf circumference (<31 cm/≥31 cm)	0.82 (0.54–1.11)	0.0004
Educational level (Below/Over mean value)	1.17 (0.56–1.83)	0.1539
Financial status (Low or medium/High)	1.12 (0.52–1.89)	0.2270
Living status (Alone/With others)	1.58 (0.83–2.26)	0.1808
Smoking habits (No/Yes)	0.83 (0.34–1.44)	0.2239
HRQOL (Low/High)	2.12 (1.84–2.59)	0.0011
IPAQ (Low/Moderate or High)	1.82 (1.49–2.28)	0.0135
PSQI (Inadequate/Adequate)	2.11 (1.72–2.48)	0.0202

HR: Hazard Ratio. CI: Confidence Interval.

## Data Availability

Not applicable.
